# Adaptive Memory: Evaluating Alternative Forms of Fitness-Relevant Processing in the Survival Processing Paradigm

**DOI:** 10.1371/journal.pone.0060868

**Published:** 2013-04-09

**Authors:** Joshua Sandry, David Trafimow, Michael J. Marks, Stephen Rice

**Affiliations:** Department of Psychology, New Mexico State University, Las Cruces, New Mexico, United States of America; Spanish National Research Council (CSIC), Spain

## Abstract

Memory may have evolved to preserve information processed in terms of its fitness-relevance. Based on the assumption that the human mind comprises different fitness-relevant adaptive mechanisms contributing to survival and reproductive success, we compared alternative fitness-relevant processing scenarios with survival processing. Participants rated words for relevancy to fitness-relevant and control conditions followed by a delay and surprise recall test (Experiment 1a). Participants recalled more words processed for their relevance to a survival situation. We replicated these findings in an online study (Experiment 2) and a study using revised fitness-relevant scenarios (Experiment 3). Across all experiments, we did not find a mnemonic benefit for alternative fitness-relevant processing scenarios, questioning assumptions associated with an evolutionary account of remembering. Based on these results, fitness-relevance seems to be too wide-ranging of a construct to account for the memory findings associated with survival processing. We propose that memory may be hierarchically sensitive to fitness-relevant processing instructions. We encourage future researchers to investigate the underlying mechanisms responsible for survival processing effects and work toward developing a taxonomy of adaptive memory.

## Introduction

A recent trend has emerged in memory research whereby scientists study memory from a functional perspective. The survival processing paradigm popularized by Nairne and colleagues [1] originated to avoid criticisms of evolutionary psychology such as post-hoc explanation and *just so* storytelling [2], [3]. One assumption of these researchers is that “[…] as products of natural selection, these systems likely bear the specific imprint of nature’s criterion–the enhancement of fitness (survival en route to differential reproduction). As a result, the ability to learn and remember will likely be influenced by the *fitness-relevance* [emphasis added] of the information and tasks involved” (p. 1–2, [4]; see also, [1], [5], [6], NSF Award # 0843165). The survival encoding strategy leads to excellent retention [1], [6]–[12] and these findings have been corroborated and extended by other labs [13]–[17]. It appears unlikely that the memorial potency of survival processing can be sufficiently explained by a mediating factor of emotional arousal [15], [16] or stereotype activation [18].

There has yet to be a large-scale test of the fitness-relevance assumption, one aimed at investigating processing benefits for a variety of fitness-relevant scenarios and then comparing the recall output in these conditions to the recall output in a traditional survival processing scenario as well as the recall output in non-fitness-relevant controls. The goal of the present research is to specifically evaluate the necessity of the *fitness-relevance* explanation for the survival processing paradigm. First, we review a number of fitness-relevant adaptive mechanisms that evolutionary psychologists suggest contributed to our survival and reproduction over evolutionary history. Next, we present a series of experiments to demonstrate that fitness-relevance is useful to an extent, but the relationship between memory and fitness-relevance may be better viewed as a continuum. Finally we discuss the implications of our findings for the theory of Adaptive Memory.

Intuitively, viewing memory from an adaptive perspective seems practical; not many scientists would disagree that human beings evolved or that we are a product of our own evolution. In fact, we agree with this basic premise and do not wish to argue against it. Our objection lies with the *all-encompassing* view of fitness-relevancy acting as a larger mechanism driving the development of human memory during the Pleistocene (i.e., environment of evolutionary adaptedness, EEA, see [19], [20]). By definition, any adaptive mechanisms would have been selected primarily for their fitness-value; that is, survival or reproductive value (see, [21] for a discussion of exaptations and spandrels). However, not all things associated with fitness (e.g., survival and reproduction) *are* necessarily associated with memory. Holistic appeals to fitness-relevance would include all adaptive mechanisms, some of which may not necessarily be associated with memory.

Assumptions related to survival processing are somewhat problematic. In the original set of experiments, Narine and his colleagues [1] suggested that a memory benefit would result when the encoding context matched the demands of the environment that shaped the adaptation. Later research supported this claim, demonstrating superior memorability for information processed with respect to an ancestral compared to modern environment [10] however, additional research did not support the ancestral assumption. Specifically, the importance of the ancestral environment appears to be overstated, because the mnemonic benefit can be found using scenarios unrelated to the African savannah [22]–[27], scenarios involving zombies [28], and even scenarios involving outer-space processing [29]. Although these findings do not completely negate the importance of ancestral environments for information processing, they identify potential issues with the supporting evolutionary assumptions.

Survival processing is also largely confounded by a personal planning component. When the planning aspect of the survival task is controlled, the superior recall attenuates [25]. Interestingly, the future-planning component alone can generate extremely high levels of recall, exceeding the typical levels of recall found with survival processing [25]. Survival processing also involves a self-referent component. In past research, Nairne and colleagues [1], [7], provided evidence that survival processing was a better encoding technique than self-referential processing. However, other research [22], [24], has shown that survival processing does not statistically differ from a type of self-referent processing that encourages participants to explicitly retrieve personal episodic memories (a type of self-referential processing not tested by Nairne and colleagues). A more plausible explanation of the survival processing findings is that a combination of different memory mechanisms (e.g., planning [25], self reference [22], [24] as well as a combination of item-specific and relational processing [13]) are responsible for the survival processing advantage.

Some functionalists engaged in adaptive memory research favor the explanation that memory has been tuned to remember information processed in terms of its *fitness-relevancy* while other researchers do not. It is possible to tease these explanations apart experimentally. If fitness-relevancy mediates the memory benefit, then we will see comparable recall levels between information processed under alternative adaptive mechanisms with unique fitness-value and information processed for survival. However, if processing information under other fitness-relevant adaptive mechanisms does not result in superior recall, then this presents a problem for theories that invoke a general appeal to fitness-relevance as an explanation for the memory benefit. The fitness-relevant explanation hinges on such a direct empirical test. In the following section, we review evidence for some adaptive mechanisms that would have led to increased fitness and successful gene propagation during the EEA.

### Adaptive Mechanisms

Many problems faced humans during the EEA and adaptive problems facilitated adaptive mechanisms (also referred to as adaptive modules) that would make it more likely for genes to pass over generations [30]. A psychologically adaptive mechanism should reflect a biological mechanism; the brain should mirror the physiology of the rest of the body [20]. Comparatively, a beautifully ornamented peacock tail leads to increased chances for a peacock to sexually reproduce with a peahen [31], subsequently passing on its genes to future generations. Analogously, humans who developed fitness-relevant adaptive mechanisms would have a similar increased chance at survival and reproduction.

Presently, we test the possibility that words processed in the following fitness-relevant conditions: fear and phobia/mating/incest avoidance/cheater detection/jealousy/infidelity/status (outlined in the following subsections), will improve memory in a manner similar to survival processing, because they also have a clear fitness-value (or fitness-relevance). If this is the case, any mechanism assisting an individual in increasing fitness and gene propagation may produce a mnemonic benefit equivalent to that bestowed by survival processing. This line of reasoning is congruent with recent suggestions made by Nairne and Pandeirada [11], (also see [4]), who suggested a possible memorial advantage associated with domain specific systems, such as reproduction, cheater detection, and kin identification. Currently, it is unclear that there will be a mnemonic benefit for alternative forms of fitness-relevant processing.

#### Fear and phobia mechanism

Evidence from a fear and phobia mechanism has been garnished from an experiment investigating visual search speed with faster responses to fear-relevant stimuli than fear-irrelevant stimuli [32]. Attentional resources seem to be allocated to stimuli that may be a potential threat to survival. Neuroimaging studies have revealed increased activation in the amygdala when participants were presented with fear arousing stimuli [33]. This brain region is often implicated in responses relating to fearful stimuli [34] as well as emotional memories [35]. Functionally, it would be adaptive to remember information processed in terms of a fear and phobia mechanism in order to avoid dangerous situations in the future.

#### Mate detection mechanism

A mate detection mechanism may manifest as the ability to identify healthy mates. For example, attractiveness and symmetry are correlated with the health of an individual [36], [37], and facial symmetry is positively associated with mate value [38]. Additionally, high pathogen levels in a specific geographical region were linked to the ratings participants gave of a potential mate’s physical attractiveness, evidence that humans are attracted to physical features indicative of pathogen resistance [39]. Individuals with attractive features are more likely to mate with other individuals [40] and pass on their genes. Functionally, a mate detection mechanism could serve a mnemonic benefit because remembering information encoded under a mating context would likely result in a greater probability of finding an optimal mate.

#### Incest avoidance mechanism

During the EEA, offspring who were a product of mating within the same genetic pool, would have been less likely to survive. Those individuals who avoided incestuous relationships would have been more likely to pass on their genes. The “Westermarck hypothesis” proposes that infants who are raised in the same home from childhood are less likely to engage in sexual relationships with each other [41]. Comparatively, there is evidence for incest avoidance in several insect [42], avian, and non human mammalian species [43]. Additionally, close kin can recognize relatives via olfactory cues [44]–[46].

Disgust may also act as a mechanism that assists in the avoidance of dangerous substances [47]. During the EEA, disgust may have served as an indicator to avoid incestuous relationships [48], [49] (but see [50]). Neurological evidence reveals activation in similar brain regions when participant’s process pathogen-related acts compared to immoral acts, some of which were incestuous [51]. It is possible that the fitness-relevance associated with an incest avoidance mechanism will extend to memory. Better memory for incest related processing would increase the likelihood of identifying kin and lower the chances of mating with kin in the future.

#### Cheater detection/infidelity detection mechanism

Evolutionary theorists have suggested a mechanism for identifying cheaters [52]–[54]. Some evidence for a cheater detection mechanism comes from a paradigm using the Wason card selection task; participants performed poorly overall on the task except when the statement being evaluated involved a violation of a social contract, (i.e., cheating, [52]). Interestingly, the cheater detection mechanism seems to be highly sensitive to acts of intentional cheating and less sensitive to accidental cheating [55]. Neurological evidence shows increased activation in the amygdala when people are presented with untrustworthy faces relative to trustworthy faces [56], [57], the same area of the brain that has been linked to emotional memories [35] and fear arousal [34]. People also have a unique ability to remember faces of cheaters [58], [59], and names of cheaters [60]. It may benefit humans to remember information processed for its relevance to detecting cheaters and/or unfaithful relationships so that they could avoid similar harmful encounters in the future.

#### Jealousy mechanism

Men and women respond negatively and with heightened physiological arousal when responding to jealous situations. Men show increased physiological arousal when the situation involved sexual infidelity and women show increased physiological arousal when the situation involved emotional infidelity [61]. It may benefit humans to remember information processed in terms of jealousy, which possibly allows for the avoidance of situations that decrease reproductive fitness.

#### Status mechanism

Gaining and maintaining status would have served as an adaptive function for both men and women because high status leads to better nutrition, greater access to resources, and improved resource allocation [62], (also see [63]). High status individuals are more likely to provide more nutrients and shelter to their offspring and increase their offspring’s chances of survival than if they had limited access to resources, something that comes with low status. Buss et al. [64] found that women preferred men who had good financial prospects and high earning potential (i.e., resource allocation potential). Men seemed to be more interested in potential mates’ appearance (i.e., reproductive success, see mate selection subheading above). The higher a man’s status, the better mate choice he has. Women, on the other hand, ostensibly sought status by searching for high status mates so that their offspring would have greater access to resources. The offspring of both males and females would benefit from high status because they would have greater access to resources [65].

## Present Experiments

Evolutionary psychologists assume that humans faced many adaptive problems during the EEA, which resulted in many domain specific mechanisms common to all humans. These mechanisms assisted our ancestors during our evolutionary history, thus contemporary humans should still exhibit some of these features [20], [30]. Adaptive memory researchers have typically tested the functional aspects of memory by using a survival processing paradigm, and they have often concluded that survival processing is a special form of processing. These researchers go further to explain the findings under an evolutionary framework and describe memory as specially tuned to remember *fitness-relevant* information [1], [4]–[6]. By borrowing from the literature on evolutionary psychology we plan to further extend the functional approach of testing memory.

There are two competing hypotheses that can be tested regarding the claim that fitness-relevance is responsible for the strong encoding technique. If information processed in terms of its fitness-relevance leads to higher recall, then the fitness-relevance explanation is corroborated. Alternatively, if information processed in terms of its fitness-relevance does not lead to higher recall, then the fitness-relevance explanation is falsified [66], [67] and the fitness-relevance auxiliary hypothesis needs to be adjusted accordingly [68]–[71].

### Ethics Statement

The present research was approved by the Institutional Review Board of New Mexico State University. All participants gave written or electronic informed consent.

### Experiment 1a

Utilizing a within-participant design and adopting the methods from Nairne et al. [1], we asked participants to rate the relevancy of words to 8 evolutionarily derived situations: survival, fear and phobia, mate selection, incest avoidance, cheater detection, jealousy, infidelity, and gaining or maintaining status. We also included 2 control conditions, pleasantness ratings (the original control [1], and possibility ratings (imagine balancing the objects on your head, e.g., bizarre imagery [72]). Following a delay, all participants were given a surprise recall task.

### Methods

#### Participants

One-hundred and fifty undergraduates (88 females and 62 males) participated for partial course credit. Two participants did not follow directions and recalled scenarios instead of words. Their data were replaced prior to analysis. Mean participant age was 20.17 (*SD = *5.03) and ranged from 18 to 62 years old.

#### Materials and procedure

Stimuli were 100 random nouns (same parameters as [17]) selected from the MRC database [73] divided into 10 random lists containing 10 words each. The order of the 10 lists and the 10 processing conditions were counterbalanced using a 10×10 Graeco-Latin square design. The presentation sequence of processing conditions was also counterbalanced across participants to eliminate order effects.

Using a within-participants design ([1], Experiment 2), participants were asked to rate 10 out of the 100 words in each of the following 10 situations resulting in 100 total ratings: how relevant is this word to a(n) survival, fear and phobia, mate selection, incest avoidance, cheater detection, jealousy, infidelity, gaining or maintaining status situation as well as pleasantness and possibility ratings (see Supporting Information for scenarios). After the rating was made, the word disappeared from the screen, followed by a brief fixation cross (1 sec), and the next word.

Following five practice trials using *word i* (e.g., word 1, word 2… word 5); the computer proceeded to the main rating block containing all 10 scenarios. Ten words in each list were presented in random order. The first screen presented the rating scenario followed by a screen reiterating that the target word would only appear for 5 seconds and they should make their rating within that time. The computer cycled through all 10 rating trials and this sequence repeated for all 10 conditions.

Participants were asked to rate words individually for each scenario on a five-point scale; the 1 key indicated totally (irrelevant/unpleasant/impossible) and the 5 key indicated totally (relevant/pleasant/possible). After the rating task, participants completed an unrelated working memory task where a string of random numbers between 4 and 9 digits long was briefly presented followed by a text box where participants input the string. The delay lasted 2 minutes followed by a surprise recall task lasting 10 minutes.

### Results and Discussion

The rationale for Experiment 1a was to compare the survival scenario to various fitness-relevant scenarios. The significance level was set at *α = *.05. The recall data from all 10 processing conditions were of main interest. We also conducted a number of additional tests to determine if the survival processing phenomena could be explained by higher ratings, longer response times, or sex differences (sex differences are sometimes useful for testing evolutionary assumptions, [6], [74], [75]).

#### Recall

An ANOVA on the ten processing conditions revealed a significant main effect for the number of words recalled, *F*(9, 1341) = 6.36, *p*<.001, η*_p_^2^* = .041. We conducted planned comparisons, comparing the number of words recalled in the survival processing condition individually to the other processing conditions. Participants recalled more words in the survival condition than any of the other conditions (see Table 1). These tests demonstrate that survival processing results in better recall than any of the other fitness-relevant processing conditions or control conditions. Importantly, this is contrary to what one would expect if *fitness-relevance* was driving the effect. The recall findings suggest that *fitness-relevance* is not the fundamental mechanism associated with the heightened recall in the survival processing scenario. Alternative forms of fitness-relevant processing did not result in exceptional recall.

**Table 1:** Recall data from Experiment 1a (within participants) & Experiment 2 (between participants).

	Experiment 1a				Experiment 2			
	No. Recalled	*SD*	*df*	*p*	No. Recalled	*SD*	*df*	*p*
Survival	2.39	1.86	–	–	15.51	5.10	–	–
Fear & Phobia	1.94	1.59	149	.03	12.68	5.41	81	.016
Mate Selection	1.87	1.42	149	.006	9.05	4.20	79	.001
Incest Avoidance	1.69	1.34	149	.001	9.65	4.83	64	.001
Cheater Detection	1.45	1.38	149	.001	9.53	4.52	79	.001
Jealousy	1.69	1.56	149	.001	10.09	4.32	75	.001
Infidelity	1.53	1.37	149	.001	9.71	4.46	83	.001
Status	1.33	1.38	149	.001	9.52	4.15	85	.001
Pleasantness	1.97	1.60	149	.03	8.88	4.48	82	.001
Visualization	1.93	2.13	149	.03	13.38	4.58	70	.069

Random assignment rendered 43 participants in the Survival condition (Experiment 2). Average number of words correctly recalled, standard deviations, degrees of freedom, and p-value (comparisons are to the survival condition).

Note. *p*-values denoted as.001 are less than or equal to.001.

It is possible that participant sex could account for some of the variance in the present study (see [6]). We tested accordingly, and the interaction did not reach significance. Although it is possible that the different mechanisms we used elicited sex differences, we did not design the study for this reason. With the exception of the analyses in the next two studies, we will not discuss participant sex further. It may also be of interest to investigate recall for both high and low performers by conducting a median split on the data. Because the counterbalancing was important to the design of the study and a median split rendered the counterbalancing extremely disproportionate and unequal, we decided against conducting this analysis.

#### Ratings

An ANOVA on the ten processing conditions revealed a significant main effect for the ratings, *F*(9, 1341) = 25.70, *p*<.001, η*_p_^2^* = .147. We individually compared the ratings given in the survival condition to the other processing conditions. Ratings made in the survival condition were higher than ratings made in all of the other conditions (Table 2) except for the ratings in the pleasantness condition (*p = *.68). To determine if the ratings influenced recall, we conducted a within participants ANCOVA with ratings as covariates; ratings were not a significant covariate (all *p*’s>.16).

**Table 2:** Average Rating from Experiment 1a (within participants), Experiment 2 (between participants) & Experiment 3 (between participants).

	Experiment 1a				Experiment 2				Experiment 3			
	Rating	*SD*	*df*	*p*	Rating	*SD*	*df*	*p*	Rating	*SD*	*df*	p
Survival	3.18	0.64	149	–	3.60	0.47	–	–	3.48	0.36	–	–
Fear & Phobia	2.72	0.65	149	.001	3.02	0.45	81	.001	3.12	0.53	186	.001
Mate Selection	2.77	0.65	149	.001	2.66	0.67	79	.001	2.99	0.64	182	.001
Incest Avoidance	2.61	0.75	149	.001	2.64	0.83	64	.001	–	–	–	–
Cheater Detection	2.82	0.73	149	.001	2.86	0.81	79	.001	–	–	–	–
Jealousy	2.77	0.74	149	.001	2.50	0.71	75	.001	–	–	–	–
Infidelity	2.88	0.68	149	.001	2.58	0.71	83	.001	–	–	–	–
Status	2.83	0.63	149	.001	2.94	0.62	85	.001	–	–	–	–
Pleasantness	3.21	0.52	149	.68	3.35	0.44	82	.01	–	–	–	–
Visualization	2.52	0.67	149	.001	3.11	0.55	70	.001	–	–	–	–
Moving (Exp 3)	–	–	–	–	–	–	–	–	3.04	0.5	181	.001

Rating, standard deviations, degrees of freedom, and p-value (comparisons are to the survival condition).

Note. *p*-values denoted as.001 are less than or equal to.001.

#### Response time

Time spent processing each word has been used as a measure of mental effort devoted to processing [1]. An ANOVA on response time reached significance, *F*(9, 1341) = 6.03, *p*<.001, η*_p_^2^* = .04, revealing that ratings took longer in the Fear and Phobia, *t*(149) = 3.16, *p*<.01 and Visualization processing conditions, *t*(149) = 4.34, *p*<.001, relative to the survival processing condition. No comparisons between the survival processing and any of the other processing conditions reached conventional levels of significance (all *p*’s>.30). Because the time spent rating words is an unlikely explanation of the recall differences, this analysis will not be discussed further.

### Experiment 1b

In Experiment 1a we found that processing information for alternative fitness-relevance situations did not lead to a memory benefit typically seen when information is processed for survival. These findings suggest that *fitness-relevance* cannot be used as the explanatory mechanism for the associated memory benefit. The present findings do not rule out the possibility that survival processing is more interesting, invokes more imagery, is more emotional, is more familiar, or is more unusual than the other processing conditions [8], [18]. In order to evaluate these possibilities, we conducted a related follow-up study where we asked independent raters to evaluate each of the 10 scenarios on these respective dimensions.

### Methods

#### Participants

Forty undergraduates who did not participate in Experiment 1a were asked to participate for extra course credit.

#### Survey packet and procedure

All 10 of the processing scenarios used in Experiment 1a were compiled into a survey packet (each scenario appeared on a separate page followed by five rating dimensions). Order of scenarios was counterbalanced across participants. Questionnaires were distributed on paper in a classroom setting. The questionnaires were self paced, lasting less than 10 minutes. Participants were asked to rate the scenario on interest, imagery, emotional arousal, familiarity, and usualness (1– Not at all/Extremely *to* 5– Very/Extremely) [8].

### Results and Discussion

We analyzed each dimension using separate ANOVAs (Table 3). Follow-up analyses revealed that it is unlikely that these particular dimensions adequately account for the superior retention associated with survival processing. For example, the Infidelity and Mate selection scenarios were not rated significantly different from the Survival scenario on any of the dimensions (Bonferroni correction, *p*<.006) but recall was significantly worse in the these conditions than it was in the Survival condition.

**Table 3:** Average rating of each scenario on the different dimensions (with p-value comparisons to the survival condition), from Experiment 1b (independent raters, within participants, bonferoni correction p<.006) and Experiment 3 (between participants, bonferoni correction p<.017).

Experiment 1a												
	Interest		Imagery		Emotionality		Familiarity		Usualness		Neg/Pos	
	M	*p*	M	*p*	M	*p*	M	*p*	M	*p*	M	*p*
Survival	3.93	–	4.23	–	3.65	–	2.93	–	2.70	–	–	–
Fear & Phobia	3.48	.008	3.85	.03	3.15	.02	3.10	.41	3.05	.03	–	–
Mate Selection	3.63	.20	3.80	.04	3.45	.38	3.28	.16	2.58	.62	–	–
Incest Avoidance	3.25	.001	2.85	.001	2.55	.001	1.80	.001	4.05	.001	–	–
Cheater Detection	3.50	.02	3.68	.002	2.93	.001	2.88	.83	2.90	.37	–	–
Jealousy	3.55	.08	3.60	.004	3.45	.39	3.15	.40	2.93	.38	–	–
Infidelity	3.95	.88	3.90	.10	4.10	.05	3.00	.78	2.33	.15	–	–
Status	3.73	.35	3.93	.15	3.15	.05	3.30	.13	2.35	.21	–	–
Pleasantness	2.45	.001	3.30	.001	2.20	.001	3.05	.66	2.15	.04	–	–
Visualization	2.53	.001	4.20	.89	1.88	.001	3.33	.15	2.83	.65	–	–
*F* (9, 351) =	10.76, *p*<.001, *ηp2* = .22		6.48, *p*<.001, *ηp2* = .14		16.20, *p*<.001, *ηp2* = .29		5.78, *p*<.001, *ηp2* = .13		8.90, *p*<.001, *ηp2* = .19		–	–
											–	
**Experiment 3**												
	**M**	***p***	**M**	***p***	**M**	***p***	**M**	***p***	**M**	***p***	**M**	***p***
Survival	3.5	–	4.33	–	2.69	–	3.3	–	2.89	–	2.91	–
Fear & Phobia	3.57	–	3.98	.37	2.9	–	3.18	.98	2.49	.90	3.44	.43
Mate Selection	3.6	–	4.11	.09	2.73	–	2.78	.50	3.28	.006	3.07	.005
Moving	3.8	–	4.22	.41	2.93	–	3.29	.002	2.91	.014	3.02	.72
*F (df) = *	*F* (3, 361) = 1.65, *p* = .18, *ηp2* = .01		*F* (3, 360) = 2.67, *p* = .05, *ηp2* = .02		*F* (3, 360) = 1.61, *p* = .33, *ηp2* = .01		*F* (3, 360) = 4.46, *p*<.01, *ηp2* = .04		*F* (3, 360) = 8.14, *p*<.001, *ηp2* = .06		*F* (3, 359) = 5.90, *p*<.001, *ηp2* = .05	

F statistic, degrees of freedom, and partial eta squared for each ANOVA are presented at the bottom for both experiments, respectively. Comparisons are to the survival condition.

Note. *p*-values denoted as.001 are less than or equal to.001.

### Experiment 2

There were limitations to the design of Experiment 1a that we sought to correct in Experiment 2. The relatively low amount of recall in Experiment 1a suggests a possible floor effect, rendering interpretation of the results problematic. In Experiment 1a we used a within-participants design. It is possible that participants were not able to make the cognitive switch between the 10 processing scenarios. Although we controlled this with counterbalancing, it remains possible that there were carry-over effects. To ensure against carry-over effects, we replicated Experiment 1a with an online between-participant version, using a different set of words in Experiment 2.

Use of the Internet to conduct memory experiments is becoming more common [76]. Research has indicated that Internet studies allow for more diverse sampling than the average participant pool, are comparable in quality to laboratory methods, offer greater anonymity than person-proctored measures, and due to screening methods, are relatively free of corrupt, repeated, or otherwise tainted data [76]–[78].

### Methods

#### Participants

Three hundred and seventy two self-selected people (297 Females and 75 Males) participated in the on-line version of the experiment. The mean age was 30.83 (*SD* = 12.22) and ranged from 18 to 67 years old. The study was listed as a “Word Rating Task” on a website where people could take personality surveys; there was no mention of the surprise recall task. Participants found the site by searching or following web links. Participants were aware that personalized feedback would be given following the study, incentivizing honesty.

#### Materials and procedure

Stimuli were 32 words used in prior research [8]. The same 10 scenarios from Experiment 1a were used in Experiment 2 (see Materials S1). Participants were able to participate in the study from anywhere they could access the Internet. Participants filled out general demographic information and then proceeded to the main rating section of the experiment. The rating scenario was randomly selected (1 per participant – between participants) and presented at the top of the page. One word from the pool of 32 stimulus words was presented below the scenario with the same five-point scale as in Experiment 1a (all words were presented in a random order for each participant). After making the rating, participants clicked a box labeled “next,” and the computer progressed to the next word. This repeated for all 32 rating trials. Following the ratings, participants performed an unrelated filler survey about their attachment style that lasted approximately 10 minutes. In the final portion of the experiment, participants were asked to try and recall the words they rated earlier in the study. Participants entered one word per text box until they could not remember any more words and then clicked a box labeled “finished.” After finishing, participants received personalized feedback about their performance on the experiment. Demographic information, individual word ratings, and individual words recalled were recorded.

### Results

In Experiment 2 we sought to replicate the findings from Experiment 1a using a between-participants design. The online experiment collected IP addresses from each participant; cases featuring a duplicate IP address or incomplete data were excluded from the analysis. Similar to Experiment 1a, the recall data were of main interest but we also conducted an analysis on the ratings, and the relationship between rating and recall.

#### Recall

A one-way ANOVA on the ten processing conditions revealed a significant main effect for the number of words recalled, *F*(9, 362) = 9.31, *p*<.001, η*_p_^2^* = .19. We conducted follow-up planned comparisons individually comparing the number of words recalled in the survival condition to each of the other processing conditions. Participants recalled more words in the survival condition than any of the other processing conditions (see Table 1). The difference between the survival and visualization conditions reached marginal significance, *p = *.07. Similar to Experiment 1a, there were no interactions with participant sex.

Experiment 2 remedied the near floor performance in Experiment 1a. Because of this improved performance, we conducted further analyses comparing the fitness-relevant scenarios to the pleasantness and visualization control conditions. We corrected alpha using a Bonferroni correction (*p = *.006 both sets of simple comparisons). In the first set of simple comparisons we compared the fitness-relevant scenarios to the Pleasantness control condition. Words processed in the Survival condition, *t*(82) = 6.32, *p*<.001, and words processed in the Fear and Phobia condition, *t*(79) = 3.44, *p*<.001, were recalled better than words processed in the Pleasantness condition (Table 1). In the second set of simple comparisons, we compared the fitness-relevant condition to the visualization control condition. Recall was better for the survival condition than it was in the visualization control condition (see planned comparison in the preceding paragraph). Recall in all of the other fitness-relevant conditions was worse than recall in the Visualization condition (all *p*’s<.006) except for the Fear and Phobia condition that did not differ significantly (*p = *.57). The Fear and Phobia condition demonstrated a mnemonic advantage compared to the Pleasantness control condition, however, it did not differ from the Visualization condition.

#### Ratings

We also analyzed the ratings given to each word with a one-way ANOVA on the ten processing conditions, which revealed a significant main effect, *F*(9, 362) = 12.40, *p*<.001, η*_p_^2^* = .24. We conducted follow up analyses on the main effect using independent sample t-tests, the analyses revealed that ratings given in the Survival processing condition were higher than ratings given in any of the other conditions, all *p*’s<.01 (see Table 2). We entered the rating data as a covariate in an ANCOVA, *F*(1, 361) = 0.50, *p = *.48, η*_p_^2^* = .001; ratings were not a significant covariate.

### Discussion

The results from Experiment 2 using a between subject design and a different stimuli list corroborate the findings from Experiment 1a. We did not find a mnemonic advantage for the alternative fitness-relevant processing conditions in either of the experiments. This can be taken as evidence *against* the explanation that processing information in terms of its *fitness-relevance* will lead to a recall benefit. What seems to be a more appropriate explanation is that fitness-relevancy may affect memory as a matter of degree and not all things related to fitness will invoke an encoding advantage. The use of a between-participants design in Experiment 2 allows us to eliminate the methodological concern, that participants may not have been able to switch their mindset from one scenario to the next, eliminating any possible of carry-over effects that may have contaminated the findings in Experiment 1a. We also saw much better recall performance remedying the potential floor effect in Experiment 1a allowing us to make meaningful comparisons to the control conditions.

One potential limitation of Experiment 2 was that it was conducted completely online; it is possible that participants wrote down the words during the rating portion of the experiment. We think this is unlikely for a number of reasons. First participants were unaware that the experiment was a memory experiment so they did not anticipate having to eventually recall the words. Second, the basic effect found in Experiment 1a was replicated in Experiment 2. Third, people who participate in studies on the website the experiment was hosted on do so in order to learn about themselves. We presented participants with personalized feedback that would have likely resulted in honest responding. Finally, even if some participants chose to write down words during the rating portion of the experiment, this would have been distributed across conditions through the use of random assignment.

We found that words were rated higher in the survival condition than any of the other conditions; however, ratings did not influence recall. Instead of recruiting undergraduate participants in Experiment 2, we recruited participants online, which allowed for a more diverse sample than Experiment 1a. As far as we are aware, this is the first demonstration of a test of the survival advantage in a non-undergraduate sample outside of a laboratory setting (although see, [79], [80] for demonstrations in children). Alternative forms of fitness-relevant processing did not elicit excellent recall and this finding is problematic for theories that suggest that an association with fitness-relevance is entirely responsible for the memory benefit.

### Experiment 3

The two preceding Experiments (1a & 2) used two control conditions (Pleasantness and Possibility ratings). These particular controls may weaken the inferences that can be made. The ratings were not schematically similar to the fitness-relevant conditions. Prior survival processing experiments have used both deep processing and/or schematically similar moving scenarios as control conditions [11]. In Experiment 3 we changed the control condition to a moving scenario (Materials S1). The use of a similar *relevancy* rating task would also allow us to make meaningful comparisons. Another potential concern with the preceding experiments is that the scenarios we used may have been worded incorrectly and this could have made it more difficult to detect any fitness-relevant processing benefits.

Recently, investigators tested a richness and distinctiveness explanation for the survival advantage (cf., levels-of-processing [81]). The typical survival scenario (three distinct problems, e.g., finding food, water, and avoiding predators) was tested against a scenario that had a single survival problem (lack of water). The survival advantage disappeared when there was only one problem. Elaborative processing associated with many distinct problems leads to a mnemonic benefit over scenarios with fewer distinct problems [82]. In the scenarios from Experiments 1a and 2 we did not control for the elaborateness of processing. Some scenarios had many distinct problems whereas others had few. Additionally, it may have been problematic that some of the wording used in the original fitness-relevant scenarios may not have reflected fitness-value. For example, the mating scenario focused on the concept of finding a new sexual partner, and sexual satisfaction (two distinct problems). Originally we chose this wording because sex is the proximate cause and reproduction is the ultimate cause [83]. The proximate cause is how the behavior works and the ultimate cause is why the behavior exists [84]. It is possible that there is not a mnemonic advantage associated with proximate causes, and instead the advantage comes when the scenario focuses on the ultimate cause.

We conducted Experiment 3 in order to address these limitations. Instead of revising all of our original fitness-relevant scenarios we further developed two specific scenarios (the Fear and Phobia scenario and the Mating scenario) and we compared these to the Survival processing scenario and the Moving control condition. We chose the Fear and Phobia scenario because the results of Experiment 2 revealed that this scenario resulted in a mnemonic advantage compared to the other fitness-relevant processing conditions, when compared to the pleasantness control. We decided to further develop the mating scenario because evolutionary psychology relies largely on inclusive fitness theory [85], that is, natural and sexual selection make up the metatheory for evolutionary psychology [68] (but see [86]). One additional reason for choosing to revise the Fear and Phobia and Mating scenarios is because other researchers have suggested that from a fitness perspective, it would be important to remember information associated with a predator or a prospective mate [4], [7].

We closely matched the language of these revised scenarios with the original survival scenario (i.e., *grasslands of a foreign land*). We also included three distinct problems in each scenario to address the levels of processing explanation. Our original fear and phobia scenario included one distinct problem *identify some things that would help you stay away from snakes and spiders.* The revised version includes three distinct problems: *remain on the lookout, avoid snakes and spiders,* and *find weapons to kill snakes and spiders*. Our original mating scenario included two distinct problems *looking for a partner to have sex with* and *identifying partners who would sexually satisfy you*. We designed the revised scenario to activate the ultimate causes and we included three distinct problems: *find a partner to reproduce,* and *mate with,* and *help you raise children.* We conducted Experiment 3 between participants in a classroom setting [25], [26] and asked participants to rate their particular scenario on the five dimensions from Experiment 1b along with an additional positive/negative dimension: *How negative or positive does the scenario make you feel?* (1– *extremely negative* to 5– *extremely positive*).

### Methods

#### Participants

Three hundred fifty-nine (150 male, 209 female) undergraduates in four introductory psychology classes volunteered to participate. The mean age was 18.95 (*SD* = 1.86) and ranged from 18 to 45 years old. The experiment was administered in mass testing classroom settings, lasting less than 15 minutes.

#### Materials

Stimuli consisted of the 32 words used in Experiment 2. There were four between participant conditions; Survival, Fear, Mating, and Moving (see Materials S1). The experimental packet consisted of 5 pages. The consent form appeared on the first page. On the bottom of each page was a picture of a stop sign along with instructions that read: *Please DO NOT turn the page until you are instructed to do so*. The scenario appeared at the top of the second page, followed by 32 words presented in one random order. Each word was accompanied by a relevancy rating scale (1-*totally irrelevant* to 5-*totally relevant*). The third page consisted of a Sudoku puzzle filler task. The fourth page consisted of the following instructions: “*We would now like you to try to recall the words you rated in the first part of the study. Please write the words, one per line, in the spaces provided below. You may recall the words in any order they come to mind”* ([25], p. 17) with 32 numbered lines positioned below these instructions. The top of the final page read: *Please rate the scenario on the following 6 dimensions,* with the scenario appearing underneath, followed by the six dimension ratings (between participants, [8]) and demographic questions.

#### Procedure

Two experimenters entered the classes and informed the participants that this was a timed survey and they should not turn the page until they were instructed to do so. All packets were comingled to ensure random assignment to one of the four processing conditions. After the experimenters distributed the packets, one experimenter monitored the front of the room and gave verbal instructions; the other monitored the back of the room. After participants filled out consent forms, the experimenter asked participants to turn to the second page and begin; participants were informed that they would have 3 minutes to complete this section. After time elapsed, the experimenter repeated these instructions for the third (2 minutes), fourth (5 minutes) and fifth (1.5 minutes) pages, respectively. It appeared that all participants followed the directions.

### Results

Our goal in Experiment 3 was to address some of the limitations regarding the wording of our original scenarios. The recall data were the focus of this experiment, but we also conducted an analysis on the ratings given to each word and the relationship between rating and recall.

#### Recall

The recall data were analyzed using a one-way ANOVA on the four processing conditions which revealed a significant main effect for the number of words recalled, *F*(3, 363) = 3.60, *p*<.05, η*_p_^2^* = .03. Follow-up planned comparisons between the Survival condition and each of the other conditions revealed that participants recalled more words in the Survival condition than any of the other conditions (all *p*’s<.01) (Table 4). Schematically similar fitness-relevant scenarios matched in the number of distinct problems and language did not result in a mnemonic benefit; the survival advantage remained robust. There were no main or interactive effects of participant sex.

**Table 4:** Recall data from Experiment 3 (between participants).

	Experiment 3			
	No. Recalled	SD	*df*	*p*
Survival	14.69	3.18	–	–
Fear & Phobia	13.37	4.06	186	.014
Mate Selection	13.13	3.87	182	.003
Moving	13.34	3.31	181	.005

Average number of words correctly recalled, standard deviations, degrees of freedom, and p-value (comparisons are to the survival condition).

#### Ratings

**Figure 1:**
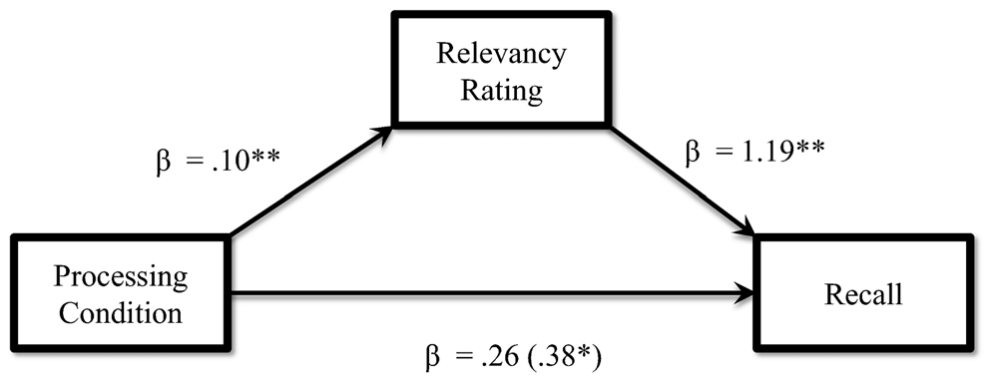
Mediation model to account for ratings in Experiment 3. Note: Direct effect (R2 = .01) and indirect effect through the mediator (R2 = .04). Coefficient presented in parentheses indicates direct effect. Note: *p<.05, **p<.001.

The rating data were analyzed using a one-way ANOVA on the four processing conditions. The analysis revealed a significant main effect for the ratings, *F*(3, 363) = 17.58, p<.001, η*_p_^2^* = .13. We conducted follow up analyses on the main effect using independent sample t-tests, the analyses revealed that ratings given in the Survival processing condition were significantly higher than ratings given in any of the other conditions (Table 2). We entered the rating data as a covariate in an ANCOVA. Results revealed that ratings may have influenced the number of words recalled, *F*(1, 362) = 7.93, *p*<.01, η*_p_^2^* = .02. In order to further investigate the impact of the relevancy ratings on the number of words recalled we conducted a mediation analysis (see Figure 1) and found ratings mediated recall, Sobel test *z = *2.61, *p*<.01. The issue of congruency will be addressed further in the General Discussion.

#### Dimensions

In Experiment 3 we were also interested in how the four processing conditions differed with respect to different dimensions. Four participants (n = 2 and n = 2, Mating and Survival, respectively) did not complete all of the ratings. We used an ANOVA on each of the individual dimensions. Four of the dimensions, Imagery, Familiarity, Usualness, and Positivity/Negativity reached significance (see all statistical analyses, Table 3). We draw similar conclusions to Experiment 1b from these analyses. For example, the Fear scenario was not rated significantly different from the Survival scenario on any of the dimensions (Bonferroni correction, *p = *.017) but there was no memory benefit when words were processed in terms of the Fear scenario. Additionally, the Mate selection scenario was rated as significantly more unusual and more positive than the Survival scenario, but this did not result in better recall.

### Discussion

In Experiment 3 we selected the two of the scenarios most likely to reveal an evolutionary advantage given the strong connection with fitness-relevance; Fear and Phobia, and Mate selection. These specific mechanisms resulted in high recall output in the first two experiments (although they did not exceed the original survival scenario) and other theorists have suggested they may be important for memory [4], [7]. Based on new research that shows a richness and distinctiveness effect related to the survival scenario [82], we revised the language and made the scenarios equal in the number of distinct problems, three in each scenario. There may have been a limitation related to the wording of our scenarios in Experiment 1a and 2, that is, the scenarios we used may not have tapped their putative fitness-relevant component. This is inevitably an inherit limitation of evolutionary psychology; there are no fossilized memory traces [5] so the design of the proper fitness-relevant scenario is largely at the discretion of existing theory. We revised the wording of the Mate selection scenario to better reflect ultimate fitness-relevancy. Finally, we used a schematically similar moving control condition in order to make more meaningful comparisons across processing conditions. With all of these additional revisions to the scenarios, we did not find a mnemonic advantage associated with these fitness-relevant scenarios. The functional suggestion that it would be important to remember information associated with a predator or a prospective mate [4], [7], was not supported in this instance. The fitness-relevant explanation of survival processing seems to be inadequate, considering these additional adaptive mechanisms (with their own unique fitness-value) did not produce excellent recall**.**

## General Discussion

Many researchers have found that processing words for survival results in better recall of those words on a following surprise retention test ([5], [9] for reviews). This effect has been explained as an evolved adaptation to remember information processed in terms of *fitness-relevance* [1], [4], [5]. In the present experiments we tested the importance of fitness-relevance as an explanation for the superior recall. There are many different adaptive mechanisms evolutionary psychologists have identified as contributing to an organism’s fitness. We tested seven of them against the original survival processing scenario along with two standard deep-processing control conditions (Experiments 1a & 2) and revised fitness-relevant scenarios and a schematically similar control condition (Experiment 3). The data consistently did not support a mnemonic advantage for information processed in alternative fitness-relevant processing conditions. The present data suggest that a general appeal directed at fitness-relevancy cannot be used as an explanatory mechanism for the memory benefit associated with survival processing. These findings require adjustment of the fitness-relevance auxiliary hypothesis and should give researchers pause, resulting in revisions to the middle-level theory of Adaptive Memory [62], [65], [69]–[71], [87]. A more accurate interpretation of the present data may be that memory is sensitive to processing information in terms of fitness-relevancy at a hierarchical level with some adaptive mechanisms having closer ties to human memory systems than other adaptive mechanisms.

In summary of the present research, we found the survival scenario outperformed the alternative fitness-relevant scenarios in encoding strength. When we removed the control conditions to focus directly on the fitness-relevant scenarios, the Fear and Phobia scenario demonstrated the next highest memory output. This was followed by some arrangement of the other conditions. Perhaps the fitness-relevant Fear and Phobia and Survival conditions activated more neural regions associated with memory than the other scenarios did, with survival processing activating a substantially larger network of brain areas that are associated with memory. In general, these findings suggest that memory is sensitive to fitness-relevant information in a continuous manor, rather than some all-or-none fitness-relevant dichotomy. Researchers have suggested that processing information in terms of its fitness-relevancy would lead to strong learning and memory (see [4] for a strong argument in favor of fitness-relevancy) but the actual strength of the association and impact on these systems was never identified.

Making the word “survival” explicit also cannot account for the memory advantage. One difference between the survival scenario and the alternative fitness-relevant scenarios was that the survival scenario used the term “survival” explicitly and the other scenarios did not mention the term “survival”. An argument could be made against the present findings that the actual word “survival” is the reason why the original scenario results in superior recall – some type of explicit awareness of survival is necessary for the performance boost. In an additional experiment, we compared the original survival scenario with an identical version of the scenario; however, the term “survival” was removed. We found no difference between the Survival-Present (*M = *14.43, *SD = *4.21) and Survival-Absent (*M = *14.64, *SD = *3.93) conditions, *t*(160) = 0.33, *p* = .74, d = .05, 95% CI [−1.05, 1.47]. The inclusion of the term itself cannot account well for the strong processing advantage and high recall output.

If it was the case that fitness-relevancy was the all-encompassing reason for the memory advantage, then other adaptive mechanisms with a clear relationship to fitness-relevancy should have demonstrated a similar processing benefit. Adaptive Memory researchers should further test this revised assumption that there is a fitness-relevancy continuum regarding memory’s sensitivity to fitness-relevant information. Although evolutionary psychology has taken somewhat of a divergent path from its older relative, evolutionary biology [86], it may be useful for a next direction of Adaptive Memory research to be the proposition of a classification or taxonomic system with theories derived to account for what selection pressures would have shaped a long-term memory system. The same taxonomic approach may also be useful when applied to other memory and learning systems. This would still avoid criticisms of just so story telling while providing a useful framework to use when testing evolutionary hypotheses directed at memory and learning.

Going further, contradictory findings have emerged in other labs regarding the necessity of a match between the encoding context and the ancestral environment where the adaptation developed. To reiterate, recent research has demonstrated that processing information in terms of threatening zombies resulted in a mnemonic advantage over proposed ancestral evolutionary predator scenarios [28]. Other studies have demonstrated that processing words in terms of alternative non-ancestral survival contexts (e.g., outer space, [29] see also, [23]) results in equivalent recall to information processed in ostensible ancestral contexts. It seems that ancestral relevance is an unlikely explanation for the superior recall ability associated with survival processing. Based on the present research, complete appeals to fitness-relevance can be ruled out as the full explanation for the powerful information processing effect. Some researchers have also identified boundary conditions where survival processing does not work.

One recent study did not find an effect of survival processing when the to-be-remembered information was faces rather than word lists [88]. Tasks requiring implicit processing of words have also failed to show a survival processing advantage [89]. Researchers have found that both true memories and false memories increase with survival processing and some consider this to be an adaptive function [14], [90]. Interestingly, when the total output is taken into account via net accuracy scores, the survival recall advantage attenuates [80]. Moreover, an additional recent study suggests that the survival advantage is not mediated by cortisol levels resultant from high stress [91].

The survival processing scenario differed from the other processing scenarios on a number of dimensions and this difference went in both directions, some scenarios were rated higher and some lower. We conclude that it is unlikely that these six dimensions: interest, imagery, emotional arousal, familiarity, unusualness, and positivity/negativity, account well for the recall advantage engendered by survival processing. This finding is consistent with similar studies conducted in other labs [8], [18].

The present findings from Experiment 3 inform the debate about whether survival processing can be explained by memory congruency effects [92], [93] (see [10] for an alternative account) but did not directly test it. We found ratings for words in the survival condition were higher than ratings in the other conditions. When we entered ratings as a covariate in Experiments 1a and 2, the Survival advantage remained robust. This was not the case in Experiment 3; ratings mediated the number of words recalled. This is not the first time that this pattern has emerged [8]. What is especially pertinent is that the word list we used in Experiment 2 and 3 was taken from a prior study investigating survival processing [8]. There was no covariance between ratings and recall in Experiment 2, however, there was in Experiment 3. The between experiments differences may have been a result of sample size, the number of participants recruited in Experiment 3 was nearly double per condition, compared to the number of participants recruited per condition in Experiment 2. We suggest this pattern of results be interpreted with caution because the analysis was post-hoc [1]. When congruency was manipulated experimentally, Nairne and Pandeirada [8] concluded that Butler et al.’s [92] findings were an artifact of their experimental design. The present study does not conclusively answer the question of congruity and this particular study was not designed to test this explanation.

One observation worth noting regarding the present manuscript is related to the effect sizes in the current studies. We report partial eta squares of.04 (Experiments 1),.19 (Experiment 2) and.03 (Experiment 3). The effect sizes could be viewed as being somewhat low, however, we believe that these effect sizes are not out of line with some of the adaptive memory literature. Some of the studies investigating survival processing have reported medium effects while others report effect sizes similar in size to those we report. Nairne et al. [1] reported partial eta square's of.09 (Experiment 1) and.17 (Experiment 3). Another lab reported a partial eta squared of.08 in their zombie experiment [28]. We suggest that as newer scenarios are developed that are closely matched to the original survival processing scenario (potentially closer in relation to survival processing on the fitness-relevance hierarchy) the effect sizes will continue to grow ever smaller, especially once the underlying mechanisms have been identified and matched across processing tasks.

The current study differs from prior research for various reasons. First, we considered and tested a variety of fitness-relevant adaptive mechanisms drawn from evolutionary psychology. This is a direct test of the fitness-relevance assumption [1], [4]–[6] and something that has been lacking in the existing survival processing literature. Second, the findings suggest that there is something about survival processing that is not captured by the other fitness-relevant mechanisms that we tested, whether it is the simultaneous activation of multiple mechanisms or something else remains an open question. The survival scenario may be more global than the other scenarios, meaning that survival processing invokes multiple adaptive mechanisms simultaneously. If these mechanisms independently activate subparts of our memory systems, it is possible that activating them in parallel (as may be the case with the survival scenario) would result in stronger memory traces. This perspective could be used as the foundation of a taxonomic structure of Adaptive Memory, whereby different mechanisms are mapped onto the taxonomy based on how much they would have been involved in the development of a memory system. In Experiment 2 and 3 we used a word list that differed from Experiment 1a and an online sample in Experiment 2, recruiting a diverse selection of participants, thus increasing the generalizability of our findings. Experiment 3 addressed some concerns regarding the control scenario and the wording of the alternative fitness-relevant scenarios. Over all of the Experiments, the survival recall advantage outperformed the alternative fitness-relevant processing scenarios; falsifying the claim that fitness-relevance mediates the effect of survival processing.

A substantial amount of fallout from the survival processing paradigm has led to investigations of what additional variables interact with, or what additional variables are affected by survival processing. At this point in time, it seems likely that research will continue in this direction; survival processing effects may be extended to other cognitive or perceptual systems. Perhaps survival processing will narrow attention, or enhance auditory perception. At the same time, an untested claim would be that survival processing will affect visual perception (see [94] for research heading in this direction). These postulated findings will be of empirical interest but will not bring us closer to understanding the underlying mechanism responsible for the effect, especially if a larger theory and a vast amount of evidence is in fact what researchers *are* actually interested in [95]. Similar to Klein [24], we believe that there is more to be gained from investigating the underlying mechanism responsible for the interesting survival processing effect (recently, Howe and Otgaar [96] also encouraged researchers to investigate the proximate mechanisms associated with adaptive memory and survival processing). We also urge researchers interested in the functional approach to veer in this direction as well as the direction of compiling a taxonomy of fitness-relevant processing effects on memory. Certainly this type of classification system will take time to produce; after all, evolutionary biologist and naturalists have been working on this type of system since the time of Aristotle. This type of focus may align evolutionary psychology with evolutionary biology, leading to richer interdisciplinary predictions [86]. It seems equally important to incorporate neurological findings in the taxonomic model in order to identify common neural substrates. What is especially important when considering these alternative underlying memory mechanisms is that fitness-relevance is not an independently useful explanation.

### Conclusion

A number of recent studies have found free recall output levels comparable to survival processing without the original survival processing scenario [22]–[29]. One explanation for the survival processing effect has been that memory may be tuned to remember information processed in terms of its fitness-relevancy [1], [4], [5]. If this assertion was correct, the current findings would have demonstrated a mnemonic benefit for the alternative fitness-relevant processing scenarios. This was not the case, the fact that the alternative fitness-relevant adaptive mechanism did not result in better memory presents a problem for explanations that suggest fitness-relevance mediates the survival processing phenomena. Fitness-relevance is too large of a construct to explain the memory findings – encompassing every evolutionary pressure that could have possibly impacted humans, even pressures that may not have directly shaped memory. The findings from the present study, along with the findings from other studies that identified boundary conditions – especially with regard to evolutionary theorizing, make a strong request to belay the current explanation and generate a revised and more comprehensive account of adaptive memory. The focus it seems should be removed from fitness-relevance as the main explanation and instead directed toward testing alternative underlying mechanisms, explanations, and identifying a taxonomic relationship between memory and adaptation. All things fitness related are not necessarily memory related and it may be useful to take cues from evolutionary biology and identify a taxonomic structure relating adaptation to memory.

## Supporting Information

Materials S1 **Processing Scenarios used in Experiments 1a, 2, and 3.**
(DOCX)
