# Supplementary material for: Adaptive Memory: Evaluating Alternative Forms of Fitness-Relevant Processing in the Survival Processing Paradigm
Source: PLoS One. 2013 Apr 9;8(4):e60868. doi: 10.1371/journal.pone.0060868 (PMC3621967; doi:10.1371/journal.pone.0060868)
Supplement: Materials S1 — Processing Scenarios used in Experiments 1a, 2, and 3. (DOCX) [file pone.0060868.s001.docx]

Supporting Materials. *Processing Scenarios used in Experiments 1a, 2, & 3*

|  |  |  |  |  |  |  |  |  |  |  |
| --- | --- | --- | --- | --- | --- | --- | --- | --- | --- | --- |
| *Experiment 1a & Experiment 2* | | | | | | | | | | |
|  |  |  |  |  |  |  |  |  |  |  |
| *Survival* | In this task, we would like you to imagine that you are stranded in the grassland of a foreign land, without any basic survival materials. Over the next few months, you'll need to find steady supplies of food and water and protect yourself from predators. We are going to show you a list of words, and we would like you to rate how relevant each of these words would be for you in this survival situation. Some of the words may be relevant and others may not-it's up to you to decide (Nairne et al. 2007). | | | | | | | | | |
|  |  |  |  |  |  |  |  |  |  |  |
|  |  |  |  |  |  |  |  |  |  |  |
|  |  |  |  |  |  |  |  |  |  |  |
| *Fear and Phobia* | In this task, we would like you to imagine that you are stranded in the grassland of a foreign land; you have a phobia (significant fear) of snakes and spiders. Over the next few months, you'll need to identify some things that would help you stay away from snakes and spiders. We are going to show you a list of words and we would like you to rate how relevant or irrelevant each of these words would be for you in this snake and spider avoidance situation. Some of the words may be relevant and others may not-it's up to you to decide. | | | | | | | | | |
|  |  |  |  |  |  |  |  |  |  |  |
|  |  |  |  |  |  |  |  |  |  |  |
|  |  |  |  |  |  |  |  |  |  |  |
| *Mate Selection* | In this task, we would like you to imagine that you are looking for a new partner to have sex with. It is important to identify partners who would sexually satisfy you. We are going to show you a list of words, and we would like you to rate how relevant each of these words would be for you in your search for a sex partner. Some of the words may be relevant and others may not-it's up to you to decide. | | | | | | | | | |
|  |  |  |  |  |  |  |  |  |  |  |
|  |  |  |  |  |  |  |  |  |  |  |
| *Incest Avoidance* | In this task, we would like you to imagine that you are looking for a new sexual partner. You may or may not be related to the people you encounter. It is extremely important to identify any potential relatives so that you can avoid incest. We are going to show you a list of words, and we would like you to rate how relevant each of these words would be for you in this incest avoidance situation. Some of the words may be relevant and others may not-it's up to you to decide. | | | | | | | | | |
|  |  |  |  |  |  |  |  |  |  |  |
|  |  |  |  |  |  |  |  |  |  |  |
|  |  |  |  |  |  |  |  |  |  |  |
| *Cheater Detection* | In this task, we would like you to imagine that you are in the grassland of a foreign land, without any friends. Over the next few months, you'll need to find people who are of good moral character (honest and loyal) and create friendships with them. It is important to be able to identify any cheaters that could cause trouble for you. We are going to show you a list of words and we would like you to rate how relevant or irrelevant each of these words would be for you in this moral judgment/cheater detection situation. Some of the words may be relevant and others may not-it's up to you to decide. | | | | | | | | | |
|  |  |  |  |  |  |  |  |  |  |  |
|  |  |  |  |  |  |  |  |  |  |  |
|  |  |  |  |  |  |  |  |  |  |  |
| *Jealousy* | In this task, we would like you to imagine yourself as an extremely jealous person. You and your significant other have relocated to a new city. Over the next few months, while you are getting used to your new location you will have to identify people who are potential rivals of your significant other. We are going to show you a list of words, and we would like you to rate how relevant each of these words would be for you in this jealousy situation. Some of the words may be relevant and others may not-it's up to you to decide. | | | | | | | | | |
|  |  |  |  |  |  |  |  |  |  |  |
|  |  |  |  |  |  |  |  |  |  |  |
|  |  |  |  |  |  |  |  |  |  |  |
| *Infidelity* | In this task, we would like you to imagine that your spouse might be having an affair. Over the next few months, you'll need to find evidence that your spouse is having an affair or is not having one. We are going to show you a list of words, and we would like you to rate how relevant each of these words would be for you in your search for evidence situation. Some of the words may be relevant and others may not-it's up to you to decide. | | | | | | | | | |
|  |  |  |  |  |  |  |  |  |  |  |
|  |  |  |  |  |  |  |  |  |  |  |
|  |  |  |  |  |  |  |  |  |  |  |
| *Status* | In this task, we would like you to imagine that you have been very successful and have achieved very high career status and very high life status. Over the next few months, you'll need to try and maintain your high status or gain an even higher status. We are going to show you a list of words, and we would like you to rate how relevant each of these words would be for you in this status-maintenance/status-gain situation. Some of the words may be relevant and others may not-it's up to you to decide | | | | | | | | | |
|  |  |  |  |  |  |  |  |  |  |  |
|  |  |  |  |  |  |  |  |  |  |  |
|  |  |  |  |  |  |  |  |  |  |  |
| *Pleasantness* | In this task, we are going to show you a list of words, and we would like you to rate the pleasantness of each word. Some of the words may be pleasant and others may not-it's up to you to decide (Nairne et al. 2007). | | | | | | | | | |
|  |  |  |  |  |  |  |  |  |  |  |
|  |  |  |  |  |  |  |  |  |  |  |
| *Visualization* | In this task, we would like you to imagine balancing an object on your head. We are going to show you a list of words, and we would like you to rate how possible it would be for you to balance that particular thing on your head. Some of the objects may be easy to balance and others may not - it's up to you to decide. | | | | | | | | | |
|  |  |  |  |  |  |  |  |  |  |  |
|  |  |  |  |  |  |  |  |  |  |  |
|  |  |  |  |  |  |  |  |  |  |  |
| *Experiment 3* | | | | | | | | | | |
|  |  |  |  |  |  |  |  |  |  |  |
| *Survival* | Same scenario as Experiment 1a & 2 except the phrase “We are going to show you a list of words” was replaced with “Below you will find a list of words” | | | | | | | | | |
|  |  |  |  |  |  |  |  |  |  |  |
| *Fear & Phobia* | In this task, we would like you to imagine that you are stranded in the grassland of a foreign land, with a significant fear of snakes and spiders. Over the next few months, you'll need to remain on the lookout and avoid snakes and spiders, and find weapons to kill snakes and spiders. *Below you will find a list of words* and we would like you to rate how relevant each of these words would be for you in this fear of snakes and spiders situation. Some of the words may be relevant and others may not—it’s up to you to decide. | | | | | | | | | |
|  |  |  |  |  |  |  |  |  |  |  |
|  |  |  |  |  |  |  |  |  |  |  |
|  |  |  |  |  |  |  |  |  |  |  |
| *Mate Selection* | In this task, we would like you to imagine that you are stranded in the grassland of a foreign land, *without* someone to mate with. Over the next few months, you’ll need to find a partner to reproduce and mate with and help you raise children. *Below you will find a list of words* and we would like you to rate how relevant each of these words would be for you in this mating situation. Some of the words may be relevant and others may not—it’s up to you to decide | | | | | | | | | |
|  |  |  |  |  |  |  |  |  |  |  |
|  |  |  |  |  |  |  |  |  |  |  |
|  |  |  |  |  |  |  |  |  |  |  |
| *Moving* | In this task, we would like you to imagine that you are planning to move to a new home in a foreign land. Over the next few months, you’ll need to locate and purchase a new home and transport your belongings. *Below you will find a list of words* and we would like you to rate how relevant each of these words would be for you in this moving situation. Some of the words may be relevant and others may not—it’s up to you to decide. | | | | | | | | | |
|  |  |  |  |  |  |  |  |  |  |  |
|  |  |  |  |  |  |  |  |  |  |  |
|  |  |  |  |  |  |  |  |  |  |  |
